# MiR-30 Family: A Novel Avenue for Treating Bone and Joint Diseases?

**DOI:** 10.7150/ijms.81990

**Published:** 2023-02-13

**Authors:** Jinming Huang, Yi Li, Siyi Zhu, Liqiong Wang, Lin Yang, Chengqi He

**Affiliations:** Department of Rehabilitation Medicine, West China Hospital, Sichuan University, Chengdu, China. Key Laboratory of Rehabilitation Medicine, West China Hospital, Sichuan University, Chengdu, China.

**Keywords:** metabolic bone diseases, miR-30, osteoporosis, osteoarthritis, bone tumor, vascular calcification, extracellular vesicles

## Abstract

Bone and joint diseases are a group of clinically heterogeneous diseases characterized by various bone strength disorders, bone structural defects and bone mass abnormalities. Common bone diseases include osteoporosis, skeletal dysplasia, and osteosarcoma, and common joint diseases include osteoarthritis, rheumatoid arthritis, and degenerative disc disease. all of them lead to high medical costs. The miR-30 family consists of a total of 5 members: miR-30a, miR-30b, miR-30c, miR-30d and miR-30e. Accumulating evidence has indicated that the miR-30 family may be involved in the occurrence and development of bone and joint diseases. For example, miR-30a is highly expressed in blood samples of osteoporosis patients, miR-30a/b increases in cartilage tissue of osteoarthritis patients, and lower expression of miR-30c is associated with higher malignance and shorter survival time of osteosarcoma. Mechanistically, by targeting crucial transcription factors (RUNX2, SOX9, beclin-1, etc.), the miR-30 family regulates some critical pathways of bone homeostasis (Wnt/β-Catenin, mTOR, PI3K/AKT, etc.). In view of the distinct actions of the miR-30 family on bone metabolism, we hypothesize that the miR-30 family may be a new remedy for the clinical treatment and prevention of some bone and joint diseases.

## Introduction

Bone is a hard mineralized tissue whose structure and function are maintained through homeostatic load-adaptive remodeling, resulting in a sophisticated bone microarchitecture to meet mechanical demands 1. Joints are structures where adjacent bones or bones and cartilage connect with each other, providing stability and flexibility to the body and limbs. Osteoporosis, osteoarthritis, and degenerative disc disease are the most common bone and joint diseases in the elderly, while skeletal dysplasia, osteosarcoma, and Ewing's sarcoma are prevalent among the young. All of these diseases impose a heavy economic and social burden around the world 2-5.

MicroRNAs (miRNAs) are short and conserved noncoding RNA strands that control gene expression by post-transcriptional gene silencing 6. They are known to target a variety of genes 7 and are involved in multiple biological processes, such as cell proliferation, differentiation, survival, and apoptosis 8.

**Figure 1:**
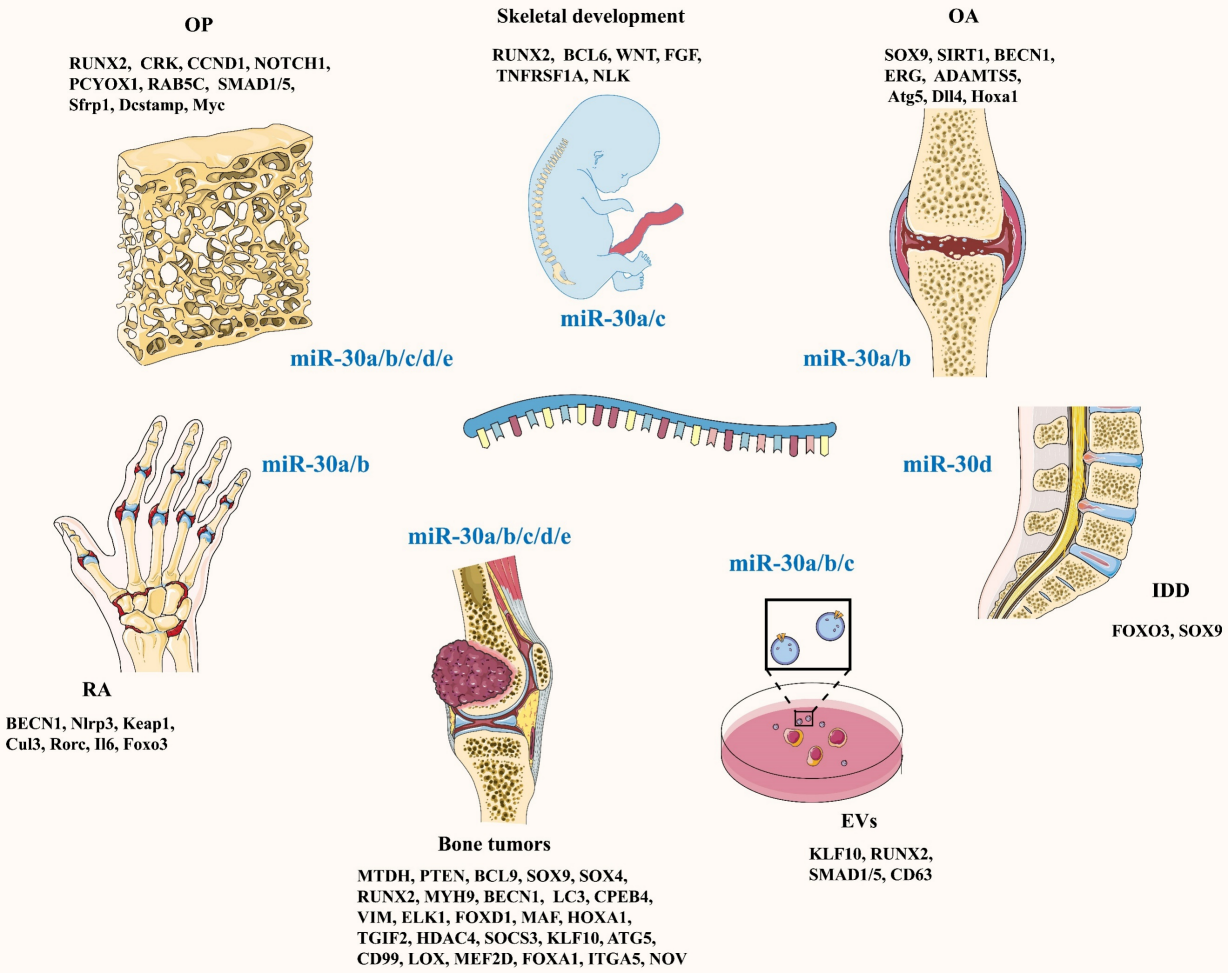
Schematic diagram of the target genes of the miR-30 family under different physiological or pathological conditions

In 2011, Rodriguez's team 9 identified miRNA-30c as an independent predictor of clinical benefits in patients with advanced breast cancer treated with tamoxifen. Since then, research on the miR-30 family has gradually increased. The miR-30 family is composed of five members: miR-30a, miR-30b, miR-30c, miR-30d and miR-30e. They are located in three chromosomal regions: 6q13 (miR-30a), 8q24.22 (miR-30b and miR-30d), and 1p34.2 (miR-30c and miR-30e) 10. To date, an increasing number of studies have shown that the miR-30 family plays a crucial role in bone formation and bone resorption through multiple pathways (**Figure 1; Table 1**), suggesting that a whole novel field of possible therapeutic schedules for bone and joint diseases is coming out.

Here, we initially reviewed the mechanism by which the miR-30 family affects skeletal development, the relationships between the miR-30 family and bone and joint diseases, as well as its therapeutic value. Finally, we discussed the effects of extracellular vesicles (EVs) containing the miR-30 family from different cell sources on bone and joint diseases and pointed out potential issues and future directions, which may be helpful for the clinical therapy of a part of bone and joint diseases.

## MiR-30 Family and Skeletal Development

The processes of osteogenesis and chondrogenesis are stringently controlled by the fate-determining transcription factor runt-related transcription factor 2 (RUNX2) 11, 12. Multiple studies have demonstrated that the miRNA-30 family is a key negative regulator of this process, targeting directly on *RUNX2* and other critical factors' gene. Zhang et al. 13 found that miR-30c targeted both *Runx2* and tricho-rhino-phalangeal syndrome I (*Trps1*) mRNA, a principal transcription factor of cartilage. Thus, miR-30c could control mesenchymal lineage progression by selectively suppressing the differentiation of osteoblasts and chondrocytes, thereby regulating skeletal development. Another study indicated that overexpression of miR-30a/b/c/d blocked BMP2-mediated osteogenic differentiation in BMSCs by targeting *Runx2* and *Smad1* mRNA, subsequently decreasing the expression and activity of alkaline phosphatase 14. Similarly, Zhang et al. 15 found miR30a was a negative regulator of BMP9-induced osteogenic differentiation by targeting *Runx2* mRNA. Additionally, miR-30b-5p inhibited the osteogenic differentiation of hBMSCs via targeting *BCL6* mRNA 16. However, contrary to most studies, one study proposed that upregulation of miR-30c could promote osteogenic differentiation by downregulating the expression of *TGIF2* and *HDAC4* mRNA 17.

Mesenchymal stem cells (MSCs) have the potential to differentiate into three lineages - in addition to the osteogenic lineage mentioned above, there are chondrogenic and adipogenic lineages. For chondrogenesis, miR-30b negatively regulates TGFβ3-induced chondrogenic differentiation of C3H10T1/2 cells by directly targeting the key cartilage differentiation factor *Sox9* mRNA 18. Specific inhibition of miR-30a/c in chondrocytes hamperes the transcription of collagen alpha-1(II) chain and Aggrecan, and subsequently decreases extracellular matrix deposition, this effect is accomplished by targeting *Snail1* mRNA, an effector that derails the normal program of permanent chondrocytes 19. In terms of adipogenesis, trough targeting *RUNX2* mRNA, miR-30 a/d not only blocked the effect of osteogenic markers and the osteogenic stemness of MSCs, but stimulated these cells to differentiate into adipocytes 20. Guo et al further explained that this phenomenon was regulated by circRNA-23525, an upstream factor of miRNA-30a 21.

The miR-30 family affects the cellular inflammatory response, but its specific role is controversial. Some scholars believe that miR-30 can inhibit the inflammatory response. Inflammasomes, such as NACHT, LRR and PYD domains-containing protein 3 (NLRP3), exert major effects in the pathogenesis of bone damage and synovitis. miR-30a negatively mediates NLRP3 expression *in vitro* by directly binding to *Nlrp3* mRNA 3ʹ UTR in TNFα-primed BMSCs, and effectively attenuates joint inflammation and bone damage in TNF^TG^ mice *in vivo* 22. Ciavarella et al. found that inflammatory stress induced by TNF-α, TGF-β1, and TGF-β3 led to endothelial-to-mesenchymal transition (End-MT), a phenotypic switch of pathological vascular changes that was associated with vascular calcification. While miR-30a-5p and miR-30d could inhibit End-MT and osteogenesis 23. Also, Yin et al found that miR-30a attenuated osteoclast formation by decreasing *Dcstamp* expression to reduce the expression of c-Fos and NFATc1 24. However, other scholars have suggested that miR-30 aggravates the inflammatory response and is detrimental to cell survival. MiR-30b could promote TNF-α induced apoptosis and enhance cartilage degradation via suppressing autophagy, and their research team detected a direct interaction between miR-30b and the mRNA 3ʹ UTRs of the autophagy genes - *Becn1* and *Atg5*, therefore reducing cellular survival during inflammation 25. Similarly, miR-30b-5p aggravated joint pain and articular cartilage loss by targeting the SIRT1-FOXO3A-mediated AILRP3 inflammasome 26. Additionally, upregulation of miR-30b in bone marrow could increase the production of IL-10 and nitric oxide by targeting *Notch1* mRNA 27.

Snail family transcriptional repressor 1 (Snail1), an effector of FGF signaling, is critical during growth plate cartilage development, but must be inhibited in the trachea to enable cartilage formation in this life-supporting organ. miR-30a/c function as direct repressors of *Snail1* expression by targeting its 3'UTR 19, which shows therapeutic potential in primary tracheomalacia.

## MiR-30 Family and Osteoporosis

Through miRNA sequencing of blood samples from postmenopausal osteoporosis (PMOP) patients, miR-30a was identified as a significantly upregulated miRNA 28. It is well known estradiol-17β (E2) is a critical regulator of bone homeostasis, promoting bone formation and reducing bone resorption. E2 was reported to suppress miR-30b expression 29, which to some extent explained the increase in plasma miR-30 in PMOP patients. Interestingly, plasma miR-30d-5p was noticeably reduced in OP patients with higher physical activity 30. Additionally, under unloading conditions, miR-30 family members were demonstrated to inhibit osteoblast differentiation by suppressing RUNX2 31.

Mechanistically, miR-30a attenuated osteoblast maturation by suppressing the expression of essential drivers of osteogenesis ‑ RUNX2 and SMAD1/5, thus inducing OP 32. Another study reported, miR-30a promoted ovariectomy-induced OP by targeting *Sfrp1* mRNA, which regulates multiple signaling pathways in osteogenic differentiation 33. Furthermore, studies have shown that several lncRNAs can downregulate miR-30 family members to influence OP development. For instance, lncRNA XIXT 34 and DGCR5 35 can upregulate RUNX2 by sponging and blocking the function of miR-30a/d, thus inducing osteogenic differentiation of hMSCs. However, there is a contrary opinion to the two previous studies - lncRNA HCG18 inhibited osteogenic differentiation of BMSCs via the miR-30a-5p/Notch1 axis. 36. One possible reason is the different cell sources, from bone marrow of PMOP patients or from femoral head tissues with or without OP. Another reason is that Che et al used hindlimb-unloaded OP mousemodel to vertify their hypothesis, while the other two studies applied serum from OP patients. Therefore, the effect of the lncRNAs/miRNA-30 family axis on osteogenesis may be affected by different OP types, sampling sites, or species, and further studies are still needed. Collectively, these findings suggest that the miR-30 family could be a possible therapeutic target to diagnose or treat OP. Further *in vivo* experiments are required to support its application.

## MiR-30 Family and Arthritis

OA is the most common chronic joint disease and is characterized by degradation or damage to articular cartilage. Recently, studies confirmed that miR-30a/b was upregulated in OA cartilage samples, which promoted OA progression by inhibiting chondrocyte proliferation and differentiation, and promoting inflammation and extracellular matrix (ECM) degradation. First, miR-30b could downregulate the mRNA expression of collagen alpha-1(II) chain and aggrecan, which are key factors for cartilage proliferation and differentiation 37. This effect was accomplished by targeting *SOX9* 38, 39 and *ERG* (ETS-related gene) mRNA 40. Second, miR-30a/b promoted the inflammatory response and ECM degradation by enhancing the expression of IL-1β during chondrogenic differentiation 41, 26. Other evidence showed that miR-30b-5p increased the protein levels of MMP-13, cleaved caspase-3 and TNF-α in chondrocytes 37. All these factors could strongly promote ECM degradation. In a rat model of chronic exercise arthritic injury, Li et al found that miR-30b-5p could regulate the inflammation, apoptosis and migration of chondrocytes by targeting *Hoxa1* mRNA 37. In addition, among the upstream genes of the miR-30 family, LncRNA LINC00461 was found to promote chondrocyte proliferation and cell cycle progression, and inhibit inflammation and ECM degradation by downregulating miR-30a-5p 42. However, Tian et al. found the opposite result in rat chondrogenic differentiation *in vitro*: miR-30a promoted chondrogenic differentiation of BMSCs by inhibiting delta-like 4 expression 43. Overall, miR-30a/b has the potential to serve as key regulator of cartilage homeostasis and potential diagnostic and a therapeutic target for OA.

In rheumatoid arthritis (RA), elevated expression of the miR-30 family contributes to anti-inflammation. In hydrogen peroxide (H_2_O_2_)-treated RA fibroblast-like synoviocytes, miR-30a-3p activated nuclear factor erythroid 2-related factor 2 (NRF2) to protect these cells against oxidative stress by targeting KEAP1 and CUL3 signaling 44. Similarly, in TNFα-primed synovial macrophages, the NLRP3 inflammasome was activated and exerted major effects on RA-mediated synovitis and bone damage, whereas microRNA-30a overexpression reversed this trend 22. In an animal model of collagen-induced arthritis, miR-30b was found to interact with *Rorc* mRNA, which encodes a protein implicated in proinflammatory Th17 cell differentiation 45. With overexpression of beclin-1 and microtubule-associated proteins light chain 3A, autophagy was increased in synovial tissue from RA patients, which was correlated with decreased levels of miR-30a 46.

## MiR-30 Family and Bone Tumors

To date, numerous studies have reported the importance of the miRNA-30 family in osteomas, including OS, ES, giant cell tumor of bone (GCT), chondrosarcoma, and other bone-eroding tumors including breast cancer metastases and multiple myeloma (MM). Among them, the most widely studied tumor is OS, and the most studied miRNA-30 family member is miR-30a.

OS, the most common malignant bone tumor, is usually found in people aged 30 years or younger. It has been reported that the miRNA-30 family is less expressed in OS tissues than in paired adjacent non-cancer tissues 47, 48. By comparing miRNAs of OS plasma samples from the Gene Expression Omnibus (GEO) database, Xu et al found that miR-30d-5p and miR-30e-5p were the central hubs of constructed miRNA-mRNA networks 49. By analyzing the relationship between the expression of miR-30c in OS tissues of different patients and the corresponding survival time, Sun et al found that lower expression of miR-30c was associated with higher malignance of OS and shorter survival time of patients. 50. Myocyte enhancer factor 2D (MEF2D) was verified to promote the initiation and progression of cancers, and Du et al indicated that miR-30a could directly target *MEF2D* mRNA to suppress OS proliferation and metastases 51. Similarly, Tao et al detected that forkhead box D1 (FOXD1) was highly expressed in OS tissues and negatively correlated with miR-30a-5p, whereas agomir-30a-5p could inhibit the proliferation, migration and invasion of OS cell lines *in vitro* 47. Other studies have reported that the miR-30 family plays a key role in the progression of OS via targeting *RUNX2* 52 and *SOX9* mRNA 53. Surprisingly, in a study of OS chemoresistance, miR-30a was confirmed to reduce chemoresistance through suppressing beclin-1-mediated autophagy 54. In recent years, studies have confirmed that noncoding RNAs interact with the miRNA-30 family to regulate the progression of OS. LncRNA 00662 upregulated the expression of ELK1 through sponging miR-30b-3p to promote the malignant behavior of OS cells 55. LncRNA MRPL23-AS1 activated the Wnt/β-Catenin signaling pathway by inhibiting miR-30b and upregulating myosin heavy chain 9 (MYH9), thereby facilitating tumor progression and carcinogenesis in OS 56. LncRNA RP11-361F15.2 was found to be highly expressed in OS tissues, which promoted CPEB4-mediated OS tumorigenesis and blocked M2-like polarization of tumor-associated macrophages by absorbing miR-30c-5p 57. LncRNA SBF2-AS1 acted as a competing endogenous RNA against miR-30a and upregulated FOXA1 expression, which contributed to proliferation, migration and invasion and inhibited apoptosis in OS cells 58. Similarly, lncRNA DICER1-AS1 promoted OS progression by the miR-30b/ATG5 axis, and knockdown of DICER1-AS1 reversed this effect 59. In addition, circular RNAs were also involved, acting in a similar manner to lncRNAs, and circ TUBGCP3 promoted progression and survivability in OS by sponging miR-30b 60. Together, overexpression of the miR-30 family can inhibit OS cell progression through multiple pathways. These studies suggested that the miR-30 family was closely related to OS progression, providing a potential possibility for the miR-30 family to be applied in the treatment and prognostic assessment of OS in the future.

ES, the second most frequent bone tumor in children and adolescents, occurs in the bones or soft tissues. Eighty-five percent of cases are characterized by a recurrent chromosome t(11;22)(q24;q12) translocation, which leads to fusions between the *EWS* and *FLI1* genes and overexpression of the EWS-FLI1 aberrant transcription factor 61; therefore, EWS-FLI1 is a critical biomarker and therapeutic target in ES. Luckly, miR-30a-5p was reported to directly connect EWS-FLI1 and effectively reduce its expression. This study also noted that mR-30a-5p could interact with CD99 membrane glycoprotein to reduce cell proliferation and invasion 62. Another study of human ES cell lines found that miR-30d blocked the cell biological progression of ES by inhibiting the MEK/ERK and PI3K/AKT pathways that are common in tumor progression. 63.

GCT, a borderline tumor with high recurrence and malignant potential, is characterized by high osteolytic activity. Compared to normal controls, miR-30c expression was obviously lower in GCT samples and cell lines, while overexpression of miR-30c suppressed cell proliferation, invasion and migration, which was achieved by targeting *HOXA1* mRNA 64. The key osteogenic transcription factor RUNX2 is also an important target of GCT progression. A study showed that miR-30a was the target of the anticancer drug - imatinib, which promoted apoptosis of GCT cells by targeting the miR-30a-mediated RUNX2 signaling pathway 65. In addition, miR-30a not only acted as a tumor suppressor, but also as a new therapeutic target for osteolysis by targeting *RUNX2* mRNA, providing more possibilities to regress GCT progression in patients 66.

In chondrosarcoma, SOX4 overexpression served as a prognostic marker in patients with low histologic grade chondrosarcoma, and miR-30a was inversely correlated with SOX4 expression in chondrosarcoma cases. Upregulating the expression of miR-30a could improve prognosis 67.

Bone is a frequently implicated organ in metastatic breast cancer, and approximately 70% of metastatic breast cancer patients suffer from bone metastases 68. miR-30 family members are involved in breast cancer bone metastases, but their roles remain controversial. Some scholars believe that miR-30 family members employ multiple mechanisms to prevent bone metastases of breast cancer. Through bioinformatics analysis and verification experiments *in vivo* and *in vitro*, Croset et al. identified many genes including osteoblastogenesis inhibition (e.g., *DKK1*), osteoclastogenesis stimulation (e.g.,* IL8, IL11*), tumor cell invasiveness (e.g., connective tissue growth factor, *ITGA5*, *ITGB3*), and bone osteomimicry (e.g., *RUNX2, CDH11*) as inhibition targets of the miR-30 family, thus impeding breast cancer bone metastases 10. Duan et al. confirmed that miR-30b inhibited the propensity of bone metastases by regulating microenvironment components. Specifically, lysyl oxidase (LOX) contributed to the remodeling of the ECM, which ultimately promoted bone metastases of breast cancer, while miR- 30b exerted an anti-bone metastatic effect by targeting LOX 69. However, other scholars believed that the miR-30 family was closely associated with the highly invasive phenotype of metastatic breast cancer. Dobson et al. indicated that miR-30c promoted the invasive phenotype via the NOV/CCN3 axis, which was completely independent of miR-30c targeting of *RUNX2* 70. By comparing the expression of miR-30 in primary tumors and paired metastatic lesions, Estevao et al found that the miR‑30b‑5p expression level was remarkably higher in bone metastasis tissue, so miR-30b-5p might indicate a higher risk of breast cancer progression 71. However, this study did not clarify the causal relationship between the increase of miR-30b-5p and bone metastasis - whether the upregulation of miR-30b-5p led to bone metastasis or bone metastasis resulted in the increase of miR-30b-5p, which requires further clarification.

MM, the second most common hematological malignant tumor, is characterized by the accumulation of abnormal monoclonal plasma cells in the bone marrow and multiple osteolytic lesions. It was reported that miR-30a-3p could downregulate the expression of its target c-Maf to inhibit bortezomib resistance in MM, while lncRNA ANGPTL1-3 abolished this effect by sponging miR-30a-3p 72. Metadherin (*MTDH*), a novel oncogene that regulates the AKT pathway, was identified as a direct target of the miR-30 family. Further data indicated that miR-30d exerted an antitumor effect by negatively regulating MTDH to inhibit the activation of the PI3K/AKT signaling pathway in U266 cells (Zhu et al., 2018). Another study using GCTB stromal cells also came to similar conclusions (Chen et al., 2018). The canonical Wnt/β-Catenin pathway is implicated in the pathogenesis of MM, while miR-30-5p functions as a MM suppressor via targeting the oncogenic Wnt/β-Catenin/BCL9 pathway 73.

Overall, the miR-30 family participates in the process of proliferation, survival, migration, and drug resistance of a variety of primary bone tumors and bone metastatic tumors by regulating the expression of their downstream target genes, which reveals the potential of miR-30 as a therapeutic target for bone-related tumors.

## MiR-30 Family and Intervertebral Disc Degeneration

Intervertebral disc degeneration (IDD) is a common cause of chronic low back pain, cervical pain, lumbar pain, and disability. After analysis of tissue samples from the degenerative lumbar nucleus pulposus, miR-30d was significantly increased in the degenerative nucleus pulposus tissue compared with the normal control group 74, 75. Mechanistically, miR-30d intensified apoptosis and extracellular matrix degradation of degenerative human nucleus pulposus cells by downregulating SOX9, thus promoting the initiation and development of IDD 74. Furthermore, another target of miR-30d in IDD was *FOXO3* mRNA, which inhibited apoptosis of nucleus pulposus cells by downregulating of *CXCL10* expression 75. The above two studies reached similar conclusions—downregulation of miRNA-30d can alleviate disc degeneration.

## MiR-30 Family and Extracellular Vesicles of Different Origins

Acting as vehicles for crosstalk between cells, EVs are secreted from various cell types. These EVs regulate other cellular biological activities by packaging and delivering active molecules including proteins, mRNA, and noncoding RNAs. BMSC-EVs can deliver noncoding RNA activated by DNA damage (NORAD) to OS cells, especially to metastatic OS tissues 82. NORAD functions as a sponge of miR-30c-5p and then upregulats the expression of Krüppel-like factor 10 (KLF10), thereby accelerating the progression and metastasis of OS 82. Patients with systemic mastocytosis (SM) usually have OP and other bone diseases due to the presence of mast cell infiltrates in bone marrow. Kim et al. found that neoplastic mast cell-derived EVs containing miR-30a blocked osteoblast differentiation and mineralization *in vitro*, and diminished osteoblast markers such as RUNX2 and SMAD1/5, trabecular bone volume, and bone microarchitecture *in vivo* 32. Calcific aortic valve disease (CAVD) is common in elderly individuals. Yang et al. revealed that telocyte-derived EVs alleviated aortic valve calcification by carrying miR-30b and then inhibited the Wnt/β-Catenin pathway 83. The study of Arntz et al. showed that bovine milk-derived EVs expressed miR-30a, exosome marker CD63, and milk-specific beta-casein and beta-lactoglobulin mRNA. Interestingly, oral administration of BMEVs attenuated OA in IL-1Ra-deficient-induced spontaneous polyarthritis and collagen-induced OA in mouse model 84.

## Conclusion

Growing evidence indicates that the miR-30 family is involved in the development of the mammalian skeletal system. However, it is noteworthy that the same miR-30 family members may play different roles in different diseases. For instance, overexpression of miR-30a in bone marrow induces OP by attenuating osteoblast maturation and intensifying the inflammatory microenvironment. Conversely, its overexpression can inhibit proliferation, migration, invasion, and survivability of OS cells, thereby delaying disease progression and improving prognosis. Additionally, the role of the miR-30 family in bone metastasis of breast cancer remains controversial.

In summary, the miR-30 family has recently been employed as a therapeutic and prognostic evaluation target for a part of bone and joint diseases. Before applying it in clinical practice, however, the following issues need our attention. First, considering its disparate roles in different diseases, before the clinical application of the miR-30 family, its pros and cons based on study evidence in different bone and joint diseases should be analyzed in detail. Second, due to the lack of relevant research, the dose-response relationship of miR-30 mimics/inhibitors for these diseases remains unknown. Third, the precise delivery of miR-30 family members to target tissues or cells to maximize their effects and reduce complications requires further research attention.

## Figures and Tables

**Figure 2:**
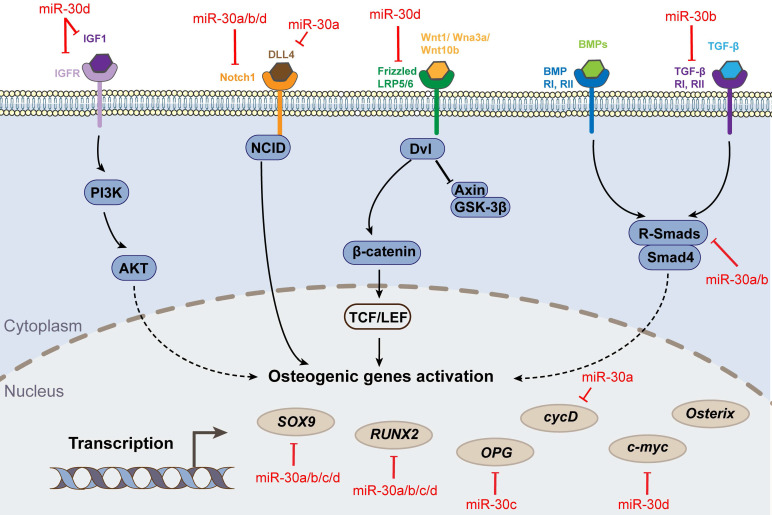
Osteogenic differentiation pathways and targets associated with miR-30 family.

**Figure 3:**
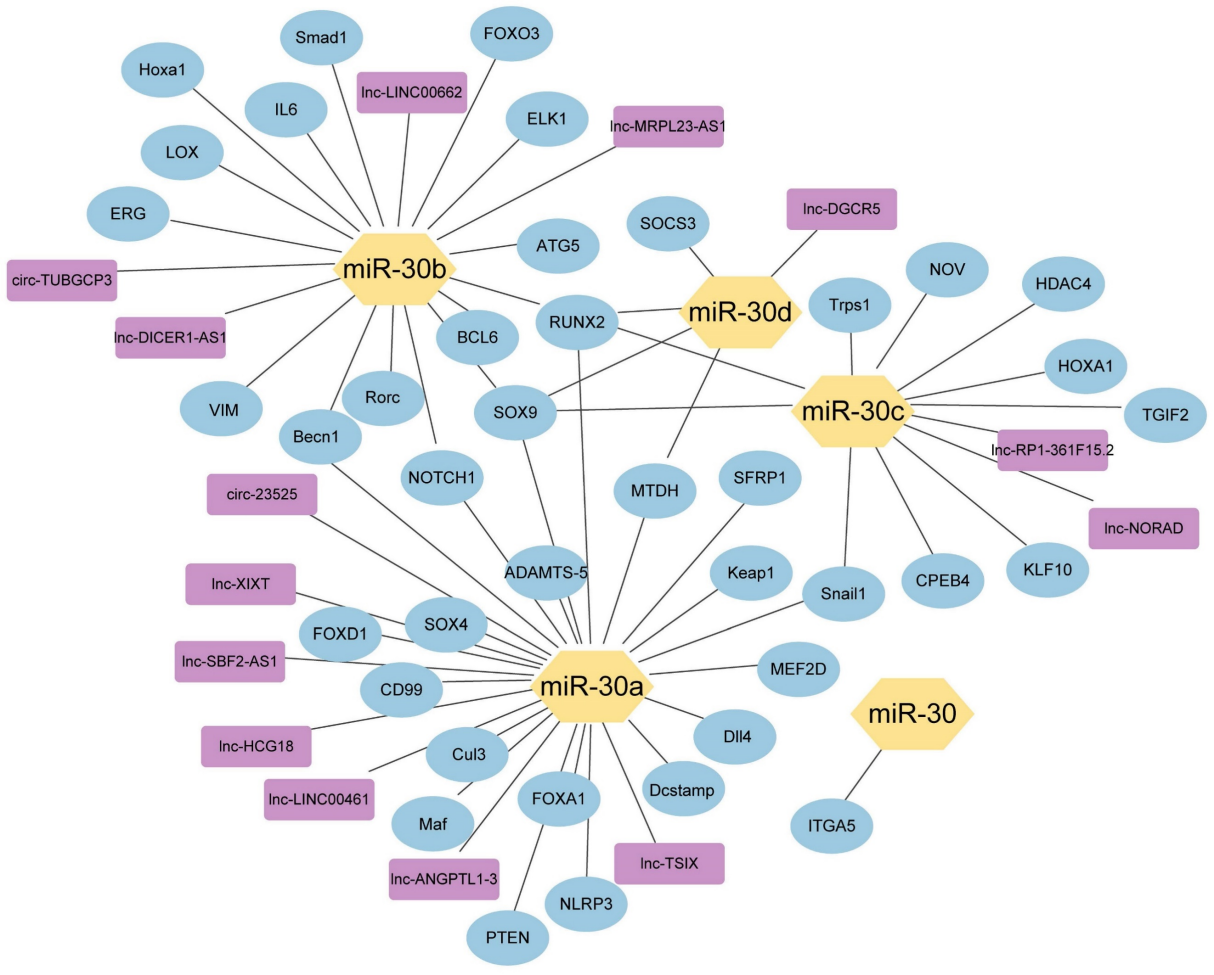
The network of miR-30 family members with their upstream genes and downstream genes. (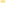) miRNA-30 family members; (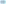) Downstream target genes verified by luciferase assay; (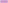) Upstream target genes verified by luciferase assay.

**Table 1:** Summary of studies investigating the regulators and effectors of the miR-30 family in bone and joint diseases.

MiRNA	Target cells/Samples/Organs	Disease or Phenotype	Intervention	Experimental Setting	Species	Target Genes	Reference
MiR-30	MC3T3-E1 cells, breast cancer lines: MDA-MB-231, T47-D, MCF-7, BT-474, ZR-751, SK-BR3, Hs-578T and MDA-B02 cells	Bone metastasis in breast cancer	Tumor xenograft	*In vivo*, *in vitro*	Human, mouse	*ITGA5, Itga5*	10
MiR-30a/b/c/d/e	MM samples, MM cell lines: H929, MM1S, and RPMI8226	MM	/	*In vivo*, *In vitro*	Human, mouse	*/*	73
MiR-30a	MG63, 143B and Saos-2 cells	OS	/	*In vitro*	Human	*PTEN*	85
MiR-30a	BMSCs, SW1353 cells	OA	/	*In vitro*	Rat	*Dll4*	43
MiR-30a	OS cell lines of 143B, MG63, and U2OS, and HS-5 cells	OS	Xenograft model	*In vivo*, *In vitro*	Human, mouse	*RUNX2, Runx2*	52
MiR-30a	HCT116, C3H10T1/2 cells	/	BMP9	*In vitro*	Mouse	*Runx2*	15
MiR-30a	MSCs	Cartilage injury	/	*In vitro*	Human	*SOX9*	39
MiR-30a	OS samples, and cells	OS	/	*In vitro*	Human	*BECN1,*	86
MiR-30a	Osteoclasts	/	/	*In vitro*	Mouse	*Dcstamp*	24
MiR-30a	Synovial samples	RA	Nlrp3^ko^/tnf^tg^	*In vivo*	NLRP3^KO^/TNF^TG^ mouse	*Nlrp3*	22
MiR-30a	Doxorubicin -resistant OS cells	OS	Doxorubicin -resistant cell line (MG63/Dox)	*In vitro*	Human	*BECN1*	54
MiR-30a	Synovial samples	RA	/	*In vivo*	Human,	*BECN1*	46
MiR 30a	Mandibular condyle	Maxillary and mandibular development	Ovariectomy	*In vivo*	Rat	*/*	87
MiR-30a	Cartilage, chondrocytes	OA	Collagenase	*In vivo*, *in vitro*	Human, rat,	*SOX9, Sox9*	38
MiR-30a	GCT stromal cells	GCT	/	*In vitro*	Human	*MTDH*	88
MiR-30a	OS smaples, cell lines: MG63, U2OS, and Saos-2 cells	OS	/	*In vitro*	Human	*MEF2D*	51
MiR-30a	HFOB1.19 and OS cell lines: Saos-2, HOS, U-2OS, SOSP9607 and MG63	OS	/	*In vitro*	Human	*FOXA1*	58
MiR-30a	OS smaples, cell lines: MG63, U2OS, and Saos-2 cells	OS	/	*In vitro*	Human	*MEF2D*	51
MiR-30a	HFOB1.19 and OS cell lines: Saos-2, HOS, U-2OS, SOSP9607 and MG63	OS	/	*In vitro*	Human	*FOXA1*	58
MiR-30a	RAW264.7, splenocytes, and intestinal cells	OA	IL-1Ra-deficient mice and collagen-induced arthritis	*In vivo*, *in vitro*	Il-1ra-/- mouse	*/*	84
MiR-30a	HFOB1.19	OP	Mastocytosis-derived EVs	*In vitro*	Human, mouse	*RUNX2, SMAD1/5; Runx2, Smad1/5*	32
MiR-30a	OA chondrocytes	OA	IL-1β	*In vitro*	Human	*ADAMTS5*	41
MiR-30a	Bone marrow-derived monocyte (BMM) cells, GCT of bone stromal cells (GCTSCs)	GCT	/	*In vitro*	Human, mouse	*RUNX2, Runx2*	66
MiR-30a	GCT cells	GCT	/	*In vitro*	Human	*RUNX2*	65
MiR-30a	Chondrosarcoma samples, SW1353 cells	Chondrosarcoma	/	*In vitro*	Human	*SOX4*	67
MiR-30a/c	Trachea	Primary tracheomalacia	COL2A1-Cre:Dicer^-/-^ mice	*In vivo*	Mouse	*Snai1*	19
MiR-30a-3p	ADSCs	/	/	*In vitro*	Mouse	*Runx2*	21
MiR-30a-3p	Serum	OP	/	*In vivo*	Human	*CRK, CCND1, PCYOX1, RAB5C*	28
MiR-30a-3p	Bone marrow, MM cell lines: NCIeH929, RPMI-8226, U266, OPM2	MM	Bortezomib	*In vivo*, *In vitro*	Human, mouse	*MAF, Maf*	72
MiR-30a-3p	RA synovial fibroblasts	RA	Freund's complete adjuvant, H_2_O_2_	*In vivo*, *In vitro*	Rat	*Keap1, Cul3*	44
MiR-30a-3p	BMSCs	OP	Ovariectomy	*In vitro*	Rat	*Sfrp1*	33
MiR-30a-5p	OS samples, U2OS and MG63 cells	OS	/	*In vivo*, *In vitro*	Human, mouse	*FOXD1, Foxd1*	47
MiR-30a-5p	OA cartilage samples, Chondrocytes	OA	TNF-α and IL-6	*In vitro*	Human	*/*	42
MiR-30a-5p	Serum, BMSCs	OP	/	*In vitro*	Human	*RUNX2*	34
MiR-30a-5p	MC3T3-E1 cells	Periodontitis	Ligature-induced Periodontitis, lipopolysaccharide	*In vivo*, *In vitro*	Rat	*Runx2*	81
MiR-30a-5p	MC3T3-E1 cells	Osteolysis	Co-Cr-Mo metal particles stimulation	*In vivo*, *in vitro*	Mouse	*Runx2*	89
MiR-30a-5p	BMSCs	OP	Hindlimb unloaded	*In vivo*, *in vitro*	Human, mouse	*NOTCH1, Notch1*	36
MiR-30a-5p	A673 cells	ES	Severe combined immuno-deficiency (SCID) mice	*In vitro*	Human, mouse	*CD99, Cd99*	62
MiR-30b/c/d/e	MC3T3-E1 cells	Disuse OP	Mechanical unloading (2D clinorotation)	*In vitro*	Mouse	*Runx2*	31
MiR-30b	OS samples, hFOB1.19 cells and OS cell lines: HOS, 143B, U2OS, MG63	OS	/	*In vitro*	Human, mouse	*MYH9, Myh9*	56
MiR-30b	OS samples, hFOB1.19, OS cell lines: HOS, 143B,U2OS, MG63	OS	/	*In vitro*	Human	*VIM*	60
MiR-30b	C3H10T1/2 cells	Cartilage defects	/	*In vitro*	Mouse	*Sox9*	18
MiR-30b	Dendritic cells, hematopoietic stem cells	/	/	*In vivo*, *In vitro*	Mouse	*Notch1*	27
MiR-30b	BMSCs	OP	Incubated with Estradiol-17β	*In vitro*	Rat	*/*	29
MiR-30b	SW1353 cells	OA	/	*In vivo*, *In vitro*	Human	*ERG*	40
MiR-30b	MG63, U2OS, HOS, 143B, Saos-2, NHOst, hFOB cells	OS	/	*In vitro*	Human	*ATG5*	59
MiR-30b	Human lung cancer cell line: 95D cells	Bone metastasis in breast cancer	Kynurenine	*In vitro*	Human	*LOX*	69
MiR-30b	Th17 cells	RA	Collagen-induced arthritis	*In vivo*, *in vitro*	Mouse	*Rorc, Il6, Foxo3*	45
MiR-30b	Unrestricted somatic stem cell	Bone defects	GSK-3β inhibitor	*In vitro*	Human	*TGFBR, NLK*	90
MiR-30b	ADTC5 cells	OA	TNF-α	*In vitro*	Mouse	*Becn1, Atg5*	25
MiR-30b-3p	OS samples, hFOB, U2OS, MG63, 143B, and HOS cells	OS	/	*In vitro*	Human	*ELK1*	55
MiR-30b-5p	Serum, osteoblasts, bone marrow monocytes	OP	*In vivo*: hindlimb unloaded, head down bedrest, and ovariectomy; *In vitro*l: M-CSF and RANKL	*In vivo*, *in vitro*	Rats, monkeys	*/*	91
MiR-30b-5p	Cancerous samples of OA patients, HC-A cells	OA	NF-κB	*In vivo*, *In vitro*	Human, rat	*SIRT1, Sirt1*	26
MiR-30b-5p	Knee joint	Chronic exercise arthritic Injury	Chronic exercise arthritic injury	*In vivo*, *In vitro*	Rat	*Hoxa1*	37
MiR-30b-5p	BMSCs	/	/	*In vitro*	Human	*BCL6*	16
MiR‑30b‑5p	Breast cancer metastatic lesions	Bone metastasis in breast cancer	/	*In vitro*	Human	*/*	71
MiR-30c	C3H10T1/2, C2C12, NIH3T3, and 3T3-L1 cells	/	/	*In vitro*	Mouse	*Trps1 and Runx2*	13
MiR-30c	MCF-7 cells, MDA-MD-231 cells	Bone metastasis in breast cancer	/	*In vitro*	Human	*NOV*	70
MiR-30c	MC3T3-E1, ATDC5, NIH3T3 cells,	/	/	*In vitro*	Mouse	*Runx2*	92
MiR-30c	OS samples, breast cancer cells	OS	Xenograft model	*In vivo*, *In vitro*	Human, mouse	*SOX9, Sox9*	53
MiR-30c	OS samples	OS	/	*In vitro*	Human	*/*	48
MiR-30c	OS samples, hFOB1.19 cells	OS	/	*In vitro*	Human	*/*	50
MiR-30c	GCT samples, breast cancer derived cell lines	GCT	/	*In vivo*, *In vitro*	Human	*HOXA1*	64
MiR-30c	MG63	OS	Nano-bioglass ceramic particles	*In vitro*	Human	*TGIF2, HDAC4*	17
MiR-30c-5p	BMSCs, 143B, HOS, Saos-2, MG63 cells	OS	/	*In vivo*, *In vitro*	Human, mouse	*KLF10, Klf10*	82
MiR-30c-5p	OS samples,	OS	Xenograft tumor model	*In vivo*, *In vitro*	Human, mouse	*CPEB4, Cpeb4*	57
MiR-30d	BMSCs	/	/	*In vitro*	Human, mouse	*WNT, FGFR, BMP, TGF, RUNX2; Wnt, Fgfr, Bmp, Tgf, Runx2;*	93
MiR-30d	Degenerative lumbar NP samples, nucleus pulposus cells	IDD	/	*In vitro*	Human	*SOX9*	74
MiR-30d	MM samples, MM cell lines: U266, H929, RPMI-8226 cells	MM	/	*In vitro*	Human	*MTDH*	94
MiR-30d	SK-ES-1 human ES cells	ES	/	*In vitro*	Human	*/*	63
MiR-30d	Degenerative lumbar nucleus pulposus samples, nucleus pulposus cells	IDD	/	*In vivo*, *In vitro*	Human	*/*	75
MiR-30d-3p	Tibia and the lumbar spine	OP	Bisphosphonate and Teriparatide	*In vivo*	Rat	*Myc*	95
MiR-30d-5p	Plasma	OP	/	*In vivo*	Human	*/*	30
MiR-30d-5p	HFOB1.19, C28/I2T	OS	/	*In vivo*, *In vitro*	Human, mouse	*SOCS3, Socs3*	96
MiR-30d-5p	Mandibular bone samples	Mandibular prognathism	/	*In vivo*	Human	*/*	97
MiR-30d-5p	MSCs	OP	/	*In vitro*	Human	*RUNX2*	35

## Abbreviations

*ADAMTS5*: ADAM metallopeptidase with thrombospondin type 1 motif 5
AKT: Akt kinase
*ATG5/Atg5*: autophagy related 5
*BCL6*: B cell leukemia/lymphoma 6
*BECN1/Becn1*: beclin-1
*BMP*/*Bmp*/BMP: bone morphogenetic protein
*CDH11*: CDH11
*Cul*/CUL3: cullin 3
*Dcstamp*: dendrocyte expressed seven transmembrane protein
*CPEB*4/*Cpeb4*/CPEB4: cytoplasmic polyadenylation element binding protein 4
*CXCL10*: C-X-C motif chemokine ligand 10
*DKK1*: dickkopf WNT signaling pathway inhibitor 1
*Dll4*: delta like canonical Notch ligand 4
E2: estradiol-17β
ECM: extracellular matrix
*ELK1*/ELK1: ETS transcription factor ELK1
End-MT: endothelial to mesenchymal transition
*ERG*: ETS-related gene
ERK: extracellular regulated MAP kinase
ES: Ewing's sarcoma
EVs: extracellular vesicles
*FGFR*: fibroblast growth factor receptor
FOS/Fos: FBJ osteosarcoma oncogene
*FOXA*1/FOXA1: forkhead box A1
FOXD1: forkhead box D1
*FOXO3*/*Foxo3*/FOXO3: forkhead box O3
FLI1: Fli-1 proto-oncogene
GCT: giant cell tumor of bone
GEO: gene expression omnibus
*HOXA1*/*Hoxa1*: homeobox A1
*HDAC4*: histone deacetylase 4
HUVECs: human umbilical vein endothelial cells
IDD: intervertebral disc degeneration
*ITGA5/Itga5*: integrin subunit alpha 5
*ITGB3*: integrin subunit beta 3
*Keap1*/KEAP1: kelch like ECH associated protein 1
*KLF10,*/*Klf10*/KLF10: Krüpppel-like factor 10
MEF2D/*MEF2D*: myocyte enhancer factor 2D
LOX: lysyl oxidase
*MAF/Maf*: MAF bZIP transcription factor
MEK: MAP kinse-ERK kinase
MM: multiple myeloma
MMP: mitochondrial membrane potential
*MTDH/*MTDH: metadherin
*Myc*: myelocytomatosis oncogene
*MYH9/ Myh9*/MYH9: myosin heavy chain 9
NFATc1: nuclear factor of activated T cells, cytoplasmic, calcineurin dependent 1
*Nlrp3/*NLRP3: NACHT, LRR and PYD domains-containing protein 3
*NLK*: nemo like kinase
NORAD: non-coding RNA activated by DNA damage
NOTCH1/*Notch1*: notch receptor 1
NRF2: nuclear factor erythroid 2-related factor 2
miRNAs: microRNAs
NRF2: nuclear factor erythroid 2-related factor 2
OA: osteoarthritis
OP: osteoporosis
OS: osteosarcoma
PMOP: postmenopausal osteoporosis
PI3K: phosphatidylinositol 3-kinase, putative
*PTEN*: phosphatase and tensin homolog
RAR: related orphan receptor gamma
*RUNX2/Runx2/*RUNX2: RUNX family transcription factor 2
*Sfrp1*: secreted frizzled related protein 1
SM: systemic mastocytosis
*Smad1/5* or SMAD1/5: SMAD family member 1/5
*Snail1/*Snail: snail family transcriptional repressor 1
SMCs: smooth muscle cells
*SIRT1/Sirt1/*SIRT1: sirtuin 1
*SOCS3/Socs3*: suppressor of cytokine signaling 3
SOX4: SRY-box transcription factor 4
*SOX9*/Sox9/SOX9: SRY-box transcription factor 9
*TGF/Tgf*: transforming growth factor
*TGFBR*: transforming growth factor beta receptor
*TGIF2*: TGFB induced factor homeobox 2
TNF-α: tumour necrosis factor alpha-like
*Trps1*/TRPS1: tricho-rhino-phalangeal syndrome I
*VIM*: vimentin

## References

[1] Berendsen AD, Olsen BR (2015). Bone development. Bone.

[2] Ayers C, Kansagara D, Lazur B, Fu R, Kwon A, Harrod C (2023). Effectiveness and Safety of Treatments to Prevent Fractures in People With Low Bone Mass or Primary Osteoporosis: A Living Systematic Review and Network Meta-analysis for the American College of Physicians. Ann Intern Med.

[3] Ferguson JL, Turner SP (2018). Bone Cancer: Diagnosis and Treatment Principles. Am Fam Physician.

[4] Folkestad L, Hald JD, Ersbøll AK, Gram J, Hermann AP, Langdahl B (2017). Fracture Rates and Fracture Sites in Patients With Osteogenesis Imperfecta: A Nationwide Register-Based Cohort Study. J Bone Miner Res.

[5] Lyu FJ, Cui H, Pan H, Mc Cheung K, Cao X, Iatridis JC (2021). Painful intervertebral disc degeneration and inflammation: from laboratory evidence to clinical interventions. Bone Res.

[6] John B, Enright AJ, Aravin A, Tuschl T, Sander C, Marks DS (2004). Human MicroRNA targets. PLoS Biol.

[7] Lewis BP, Burge CB, Bartel DP (2005). Conserved seed pairing, often flanked by adenosines, indicates that thousands of human genes are microRNA targets. Cell.

[8] Hwang HW, Mendell JT (2006). MicroRNAs in cell proliferation, cell death, and tumorigenesis. Br J Cancer.

[9] Rodriguez-Gonzalez FG, Sieuwerts AM, Smid M, Look MP, Meijer-van Gelder ME, de Weerd V (2011). MicroRNA-30c expression level is an independent predictor of clinical benefit of endocrine therapy in advanced estrogen receptor positive breast cancer. Breast Cancer Res Treat.

[10] Croset M, Pantano F, Kan CWS, Bonnelye E, Descotes F, Alix-Panabieres C (2018). miRNA-30 Family Members Inhibit Breast Cancer Invasion, Osteomimicry, and Bone Destruction by Directly Targeting Multiple Bone Metastasis-Associated Genes. Cancer Research.

[11] Franceschi RT, Xiao GZ (2003). Regulation of the osteoblast-specific transcription factor, runx2: Responsiveness to multiple signal transduction pathways. Journal of Cellular Biochemistry.

[12] Komori T (2002). Runx2, a multifunctional transcription factor in skeletal development. Journal of Cellular Biochemistry.

[13] Zhang Y, Xie R-l, Gordon J, LeBlanc K, Stein JL, Lian JB (2012). Control of Mesenchymal Lineage Progression by MicroRNAs Targeting Skeletal Gene Regulators Trps1 and Runx2. Journal of Biological Chemistry.

[14] Wu T, Zhou H, Hong Y, Li J, Jiang X, Huang H (2012). miR-30 family members negatively regulate osteoblast differentiation. J Biol Chem.

[15] Zhang R, Weng Y, Li B, Jiang Y, Yan S, He F (2015). BMP9-induced osteogenic differentiation is partially inhibited by miR-30a in the mesenchymal stem cell line C3H10T1/2. Journal of Molecular Histology.

[16] Luo Y, Zhou F, Wu X, Li Y, Ye B (2022). miR-30b-5p inhibits osteoblast differentiation through targeting BCL6. Cell Cycle.

[17] Moorthi A, Vimalraj S, Avani C, He Z, Partridge NC, Selvamurugan N (2013). Expression of microRNA-30c and its target genes in human osteoblastic cells by nano-bioglass ceramic-treatment. International Journal of Biological Macromolecules.

[18] Wa Q, He P, Huang S, Zuo J, Li X, Zhu J (2017). miR-30b regulates chondrogenic differentiation of mouse embryo-derived stem cells by targeting SOX9. Experimental and Therapeutic Medicine.

[19] Gradus B, Alon I, Hornstein E (2011). miRNAs control tracheal chondrocyte differentiation. Developmental Biology.

[20] Zaragosi LE, Wdziekonski B, Brigand KL, Villageois P, Mari B, Waldmann R (2011). Small RNA sequencing reveals miR-642a-3p as a novel adipocyte-specific microRNA and miR-30 as a key regulator of human adipogenesis. Genome Biol.

[21] Guo Z, Zhao L, Ji S, Long T, Huang Y, Ju R (2021). CircRNA-23525 regulates osteogenic differentiation of adipose-derived mesenchymal stem cells via miR-30a-3p. Cell and Tissue Research.

[22] Yang Q, Zhao W, Chen Y, Chen Y, Shi J, Qin R (2021). RelA/MicroRNA-30a/NLRP3 signal axis is involved in rheumatoid arthritis via regulating NLRP3 inflammasome in macrophages. Cell Death & Disease.

[23] Ciavarella C, Motta I, Vasuri F, Fittipaldi S, Valente S, Pollutri D (2021). Involvement of miR-30a-5p and miR-30d in Endothelial to Mesenchymal Transition and Early Osteogenic Commitment under Inflammatory Stress in HUVEC. Biomolecules.

[24] Yin Y, Tang L, Chen J, Lu X (2017). MiR-30a attenuates osteoclastogenesis via targeting DC-STAMP-c-Fos-NFATc1 signaling. American Journal of Translational Research.

[25] Chen Z, Jin T, Lu Y (2016). AntimiR-30b Inhibits TNF-alpha Mediated Apoptosis and Attenuated Cartilage Degradation through Enhancing Autophagy. Cellular Physiology and Biochemistry.

[26] Xu H, Zhang J, Shi X, Li X, Zheng C (2021). NF-kappa B-inducible miR-30b-5p aggravates joint pain and loss of articular cartilage via targeting SIRT1-FoxO3a-mediated AILRP3 inflammasome. Aging-Us.

[27] Su X, Qian C, Zhang Q, Hou J, Gu Y, Han Y (2013). miRNomes of haematopoietic stem cells and dendritic cells identify miR-30b as a regulator of Notch1. Nature Communications.

[28] Wang R, Lu A, Liu W, Yue J, Sun Q, Chen J (2020). Searching for valuable differentially expressed miRNAs in postmenopausal osteoporosis by RNA sequencing. Journal of Obstetrics and Gynaecology Research.

[29] Liu G, Lu Y, Mai Z, Liu R, Peng Z, Chen L (2019). Suppressing MicroRNA-30b by Estrogen Promotes Osteogenesis in Bone Marrow Mesenchymal Stem Cells. Stem Cells International. 2019.

[30] Kranjc T, Milojevic M, Kocjan T, Jensterle M, Marc J, Ostanek B (2020). Plasma levels of miR-30d-5p are decreased in regularly exercising postmenopausal women. Menopause-the Journal of the North American Menopause Society.

[31] Zhang L, Li G, Wang K, Wang Y, Dong J, Wang H (2020). MiR-30 family members inhibit osteoblast differentiation by suppressing Runx2 under unloading conditions in MC3T3-E1 cells. Biochemical and Biophysical Research Communications.

[32] Kim D-K, Bandara G, Cho Y-E, Komarow HD, Donahue DR, Karim B (2021). Mastocytosis-derived extracellular vesicles deliver miR-23a and miR-30a into pre-osteoblasts and prevent osteoblastogenesis and bone formation. Nature Communications.

[33] Liu HP, Hao DJ, Wang XD, Hu HM, Li YB, Dong XH (2019). MiR-30a-3p promotes ovariectomy-induced osteoporosis in rats via targeting SFRP1. European Review for Medical and Pharmacological Sciences.

[34] Zhang HL, Du XY, Dong QR (2019). LncRNA XIXT promotes osteogenic differentiation of bone mesenchymal stem cells and alleviates osteoporosis progression by targeting miRNA-30a-5p. European Review for Medical and Pharmacological Sciences.

[35] Wu Z-h, Huang K-h, Liu K, Wang G-t, Sun Q (2018). DGCR5 induces osteogenic differentiation by up-regulating Runx2 through miR-30d-5p. Biochemical and Biophysical Research Communications.

[36] Che M, Gong W, Zhao Y, Liu M (2020). Long noncoding RNA HCG18 inhibits the differentiation of human bone marrow-derived mesenchymal stem cells in osteoporosis by targeting miR-30a-5p/NOTCH1 axis. Molecular Medicine.

[37] Li M, Gai F, Chen H (2021). MiR-30b-5p Influences Chronic Exercise Arthritic Injury by Targeting Hoxa1. International Journal of Sports Medicine.

[38] Chang T, Xie J, Li H, Li D, Liu P, Hu Y (2016). MicroRNA-30a promotes extracellular matrix degradation in articular cartilage via downregulation of Sox9. Cell Proliferation.

[39] Zhang H, Wang Y, Yang G, Yu H, Zhou Z, Tang M (2019). MicroRNA-30a regulates chondrogenic differentiation of human bone marrow-derived mesenchymal stem cells through targeting Sox9. Experimental and Therapeutic Medicine.

[40] Li L, Yang C, Liu X, Yang S, Ye S, Jia J (2015). Elevated expression of microRNA-30b in osteoarthritis and its role in ERG regulation of chondrocyte. Biomedicine & Pharmacotherapy.

[41] Ji Q, Xu X, Zhang Q, Kang L, Xu Y, Zhang K (2016). The IL-1 beta/AP-1/miR-30a/ADAMTS-5 axis regulates cartilage matrix degradation in human osteoarthritis. Journal of Molecular Medicine-Jmm.

[42] Zhang Y, Ma L, Wang C, Wang L, Guo Y, Wang G (2020). Long noncoding RNA LINC00461 induced osteoarthritis progression by inhibiting miR-30a-5p. Aging-Us.

[43] Tian Y, Guo R, Shi B, Chen L, Yang L, Fu Q (2016). MicroRNA-30a promotes chondrogenic differentiation of mesenchymal stem cells through inhibiting Delta-like 4 expression. Life Sciences.

[44] Lv X, Huang J, Wang H (2021). MiR-30a-3p ameliorates oxidative stress in rheumatoid arthritis synovial fibroblasts via activation of Nrf2-ARE signaling pathway. Immunology Letters.

[45] Donate PB, Fornari TA, Macedo C, Cunha TM, Nascimento DCB, Sakamoto-Hojo ET (2013). T Cell Post-Transcriptional miRNA-mRNA Interaction Networks Identify Targets Associated with Susceptibility/Resistance to Collagen-induced Arthritis. Plos One.

[46] Xu K, Xu P, Yao J-F, Zhang Y-G, Hou W-k, Lu S-M (2013). Reduced apoptosis correlates with enhanced autophagy in synovial tissues of rheumatoid arthritis. Inflammation Research.

[47] Tao J, Cong H, Wang H, Zhang D, Liu C, Chu H (2018). MiR-30a-5p inhibits osteosarcoma cell proliferation and migration by targeting FOXD1. Biochemical and Biophysical Research Communications.

[48] Yang W, Qi Y-b, Si M, Hou Y, Nie L (2020). A comprehensive analysis for associations between multiple microRNAs and prognosis of osteosarcoma patients. Peerj.

[49] Xu K, Zhang P, Zhang J, Quan H, Wang J, Liang Y (2021). Identification of potential micro-messenger RNAs (miRNA-mRNA) interaction network of osteosarcoma. Bioengineered.

[50] Sun R, Muheremu A, Hu Y (2018). miRNA-30c can be used as a target in the diagnosis and treatment of osteosarcoma. Oncotargets and Therapy.

[51] Du L, Chen T, Zhao K, Yang D (2018). miR-30a suppresses osteosarcoma proliferation and metastasis by downregulating MEF2D expression. Oncotargets and Therapy.

[52] Zhang R, Yan S, Wang J, Deng F, Guo Y, Li Y (2016). MiR-30a regulates the proliferation, migration, and invasion of human osteosarcoma by targeting Runx2. Tumor Biology.

[53] Zhang XD, Wang YN, Feng XY, Yang JY, Ge YY, Kong WQ (2018). Biological function of microRNA-30c/SOX9 in pediatric osteosarcoma cell growth and metastasis. European Review for Medical and Pharmacological Sciences.

[54] Xu R, Liu S, Chen H, Lao L (2016). MicroRNA-30a downregulation contributes to chemoresistance of osteosarcoma cells through activating Beclin-1-mediated autophagy. Oncology Reports.

[55] Wang B, Xu Z, Wang X, Xia S, Cai P, Wang M (2022). Knockdown of lncRNA LINC00662 suppresses malignant behaviour of osteosarcoma cells via competition with miR-30b-3p to regulate ELK1 expression. Journal of Orthopaedic Surgery and Research.

[56] Zhang H, Liu S, Tang L, Ge J, Lu X (2021). Long non-coding RNA (LncRNA) MRPL23-AS1 promotes tumor progression and carcinogenesis in osteosarcoma by activating Wnt/beta-catenin signaling via inhibiting microRNA miR-30b and upregulating myosin heavy chain 9 (MYH9). Bioengineered.

[57] Yang D, Liu K, Fan L, Liang W, Xu T, Jiang W (2020). LncRNA RP11-361F15.2 promotes osteosarcoma tumorigenesis by inhibiting M2-Like polarization of tumor-associated macrophages of CPEB4. Cancer Letters.

[58] Dai J-H, Huang W-Z, Li C, Deng J, Lin S-J, Luo J (2019). Silencing of long noncoding RNA SBF2-AS1 inhibits proliferation, migration and invasion and contributes to apoptosis in osteosarcoma cells by upregulating microRNA-30a to suppress FOXA1 expression. Cell Cycle.

[59] Gu Z, Hou Z, Zheng L, Wang X, Wu L, Zhang C (2018). LncRNA DICER1-AS1 promotes the proliferation, invasion and autophagy of osteosarcoma cells via miR-30b/ATG5. Biomedicine & Pharmacotherapy.

[60] Xu Y, Yao T, Huang K, Liu G, Huang Y, Gao J (2020). Circular RNA circTUBGCP3 Is Up-Regulated and Promotes Cell Proliferation, Migration and Survivability via Sponge mir-30b in Osteosarcoma. Oncotargets and Therapy.

[61] Delattre O, Zucman J, Plougastel B, Desmaze C, Melot T, Peter M (1992). Gene fusion with an ETS DNA-binding domain caused by chromosome translocation in human tumours. Nature.

[62] Franzetti GA, Laud-Duval K, Bellanger D, Stern MH, Sastre-Garau X, Delattre O (2013). MiR-30a-5p connects EWS-FLI1 and CD99, two major therapeutic targets in Ewing tumor. Oncogene.

[63] Ye C, Yu X, Liu X, Dai M, Zhang B (2018). miR-30d inhibits cell biological progression of Ewing's sarcoma by suppressing the MEK/ERK and PI3K/Akt pathways *in vitro*. Oncology Letters.

[64] Ni LY, Zhao JD, Lu YH, Li W, Li BL, Wang XC (2017). MicroRNA-30c suppressed giant-cell tumor of bone cell metastasis and growth via targeting HOXA1. European Review for Medical and Pharmacological Sciences.

[65] Liao Y, Lv G, Wang B, Kuang L, Wang X (2016). Imatinib promotes apoptosis of giant cell tumor cells by targeting microRNA-30a-mediated runt-related transcription factor 2. Molecular Medicine Reports.

[66] Huang Q, Jiang Z, Meng T, Yin H, Wang J, Wan W (2014). MiR-30a inhibits osteolysis by targeting RunX2 in giant cell tumor of bone. Biochemical and Biophysical Research Communications.

[67] Lu N, Lin T, Wang L, Qi M, Liu Z, Dong H (2015). Association of SOX4 regulated by tumor suppressor miR-30a with poor prognosis in low-grade chondrosarcoma. Tumor Biology.

[68] Othman A, Winogradzki M, Lee L, Tandon M, Blank A, Pratap J (2021). Bone Metastatic Breast Cancer: Advances in Cell Signaling and Autophagy Related Mechanisms. Cancers (Basel).

[69] Duan Z, Li L, Li Y (2019). Involvement of miR-30b in kynurenine-mediated lysyl oxidase expression. Journal of Physiology and Biochemistry.

[70] Dobson JR, Taipaleenmaeki H, Hu Y-J, Hong D, van Wijnen AJ, Stein JL (2014). hsa-mir-30c promotes the invasive phenotype of metastatic breast cancer cells by targeting NOV/CCN3. Cancer Cell International.

[71] Estevao-Pereira H, Lobo J, Salta S, Amorim M, Lopes P, Cantante M (2019). Overexpression of circulating MiR-30b-5p identifies advanced breast cancer. Journal of Translational Medicine.

[72] Nian F, Zhu J, Chang H (2019). Long non-coding RNA ANGPTL1-3 promotes multiple myeloma bortezomib resistance by sponging miR-30a-3p to activate c-Maf expression. Biochemical and Biophysical Research Communications.

[73] Zhao J-J, Lin J, Zhu D, Wang X, Brooks D, Chen M (2014). miR-30-5p Functions as a Tumor Suppressor and Novel Therapeutic Tool by Targeting the Oncogenic Wnt/beta-Catenin/BCL9 Pathway. Cancer Research.

[74] Lv J, Li S, Wan T, Yang Y, Cheng Y, Xue R (2018). Inhibition of microRNA-30d attenuates the apoptosis and extracellular matrix degradation of degenerative human nucleus pulposus cells by up-regulating SOX9. Chemico-Biological Interactions.

[75] Xia P, Gao X, Li F, Shao L, Sun Y (2021). Down-Regulation of microRNA-30d Alleviates Intervertebral Disc Degeneration Through the Promotion of FOXO3 and Suppression of CXCL10. Calcified Tissue International.

[76] Vasuri F, Ciavarella C, Fittipaldi S, Pini R, Vacirca A, Gargiulo M (2020). Different histological types of active intraplaque calcification underlie alternative miRNA-mRNA axes in carotid atherosclerotic disease. Virchows Archiv.

[77] Zhang Q, Chen T, Zhang Y, Lyu L, Zhang B, Huang C (2021). MiR-30c-5p regulates adventitial progenitor cells differentiation to vascular smooth muscle cells through targeting OPG. Stem Cell Research & Therapy.

[78] Balderman JAF, Lee H-Y, Mahoney CE, Handy DE, White K, Annis S (2012). Bone Morphogenetic Protein-2 Decreases MicroRNA-30b and MicroRNA-30c to Promote Vascular Smooth Muscle Cell Calcification. Journal of the American Heart Association.

[79] Zhang M, Liu X, Zhang X, Song Z, Han L, He Y (2014). MicroRNA-30b is a multifunctional regulator of aortic valve interstitial cells. Journal of Thoracic and Cardiovascular Surgery.

[80] Xu T-H, Qiu X-B, Sheng Z-T, Han Y-R, Wang J, Tian B-Y (2019). Restoration of microRNA-30b expression alleviates vascular calcification through the mTOR signaling pathway and autophagy. Journal of Cellular Physiology.

[81] Liu X, Yang B, Zhang Y, Guo X, Yang Q, Liu X (2021). miR-30a-5p inhibits osteogenesis and promotes periodontitis by targeting Runx2. Bmc Oral Health.

[82] He H, Ding M, Li T, Zhao W, Zhang L, Yin P (2022). Bone mesenchymal stem cell-derived extracellular vesicles containing NORAD promote osteosarcoma by miR-30c-5p. Laboratory Investigation.

[83] Yang R, Tang Y, Chen X, Yang Y (2021). Telocytes-derived extracellular vesicles alleviate aortic valve calcification by carrying miR-30b. Esc Heart Failure.

[84] Arntz OJ, Pieters BCH, Oliveira MC, Broeren MGA, Bennink MB, de Vries M (2015). Oral administration of bovine milk derived extracellular vesicles attenuates arthritis in two mouse models. Molecular Nutrition & Food Research.

[85] Zhong B, Guo S, Zhang W, Zhang C, Wang Y, Zhang C (2017). Bioinformatics prediction of miR-30a targets and its inhibition of cell proliferation of osteosarcoma by up-regulating the expression of PTEN. Bmc Medical Genomics.

[86] Yuan T, Wang J, Wei X, Wang G, Xu N, Fan L (2016). MicroRNA-30a Inhibits Proliferation and Metastasis of Osteosarcoma Cells by Modulating Autophagy. Journal of Biobased Materials and Bioenergy.

[87] Bergamo AZN, Madalena IR, Omori MA, Ramazzotto LA, Nelson-Filho P, Baratto-Filho F (2022). Estrogen deficiency during puberty affects the expression of microRNA30a and microRNA503 in the mandibular condyle. Annals of Anatomy-Anatomischer Anzeiger.

[88] Chen F, Wang S, Wei Y, Wu J, Huang G, Chen J (2018). Norcantharidin modulates the miR-30a/Metadherin/AKT signaling axis to suppress proliferation and metastasis of stromal tumor cells in giant cell tumor of bone. Biomedicine & Pharmacotherapy.

[89] Bu Y, Zheng D, Wang L, Liu J (2018). LncRNA TSIX promotes osteoblast apoptosis in particle-induced osteolysis by down-regulating miR-30a-5p. Connective Tissue Research.

[90] Bakhshandeh B, Soleimani M, Hafizi M, Paylakhi SH, Ghaemi N (2012). MicroRNA signature associated with osteogenic lineage commitment. Molecular Biology Reports.

[91] Chen J, Li K, Pang Q, Yang C, Zhang H, Wu F (2016). Identification of suitable reference gene and biomarkers of serum miRNAs for osteoporosis. Scientific Reports.

[92] Zhang Y, Xie R-l, Croce CM, Stein JL, Lian JB, van Wijnen AJ (2011). A program of microRNAs controls osteogenic lineage progression by targeting transcription factor Runx2. Proceedings of the National Academy of Sciences of the United States of America.

[93] Eguchi T, Watanabe K, Hara ES, Ono M, Kuboki T, Calderwood SK (2013). OstemiR: A Novel Panel of MicroRNA Biomarkers in Osteoblastic and Osteocytic Differentiation from Mesencymal Stem Cells. Plos One.

[94] Zhu B, Chen H, Zhang X, Pan Y, Jing R, Shen L (2018). Serum miR-30d as a novel biomarker for multiple myeloma and its antitumor role in U266 cells through the targeting of the MTDH/PI3K/Akt signaling pathway. International Journal of Oncology.

[95] Weigl M, Kocijan R, Ferguson J, Leinfellner G, Heimel P, Feichtinger X (2021). Longitudinal Changes of Circulating miRNAs During Bisphosphonate and Teriparatide Treatment in an Animal Model of Postmenopausal Osteoporosis. Journal of Bone and Mineral Research.

[96] Hu Y, Luo X, Zhou J, Chen S, Gong M, Deng Y (2021). Piperlongumine inhibits the progression of osteosarcoma by downregulating the SOCS3/JAK2/STAT3 pathway via miR-30d-5p. Life Sciences.

[97] Tian Y, Liu J, Bai X, Tan X, Cao Y, Qin K (2015). MicroRNA expression profile of surgical removed mandibular bone tissues from patients with mandibular prognathism. Journal of Surgical Research.

